# Effects of L-Theanine on Posttraumatic Stress Disorder Induced Changes in Rat Brain Gene Expression

**DOI:** 10.1155/2014/419032

**Published:** 2014-08-03

**Authors:** Tomás Eduardo Ceremuga, Stephanie Martinson, Jason Washington, Robert Revels, Jessica Wojcicki, Damali Crawford, Robert Edwards, Joshua Luke Kemper, William Luke Townsend, Geno M. Herron, George Allen Ceremuga, Gina Padron, Michael Bentley

**Affiliations:** ^1^US Army Graduate Program in Anesthesia Nursing, AMEDDC&S, Institute of Surgical Research, Fort Sam Houston, TX 78234, USA; ^2^United States Army Graduate Program in Anesthesia Nursing, Fort Sam Houston, TX 78234, USA; ^3^The Geneva Foundation, WA 98402, USA

## Abstract

Posttraumatic stress disorder (PTSD) is characterized by the occurrence of a traumatic event that is beyond the normal range of human experience. The future of PTSD treatment may specifically target the molecular mechanisms of PTSD. In the US, approximately 20% of adults report taking herbal products to treat medical illnesses. L-theanine is the amino acid in green tea primarily responsible for relaxation effects. No studies have evaluated the potential therapeutic properties of herbal medications on gene expression in PTSD. We evaluated gene expression in PTSD-induced changes in the amygdala and hippocampus of Sprague-Dawley rats. The rats were assigned to PTSD-stressed and nonstressed groups that received either saline, midazolam, L-theanine, or L-theanine + midazolam. Amygdala and hippocampus tissue samples were analyzed for changes in gene expression. One-way ANOVA was used to detect significant difference between groups in the amygdala and hippocampus. Of 88 genes examined, 17 had a large effect size greater than 0.138. Of these, 3 genes in the hippocampus and 5 genes in the amygdala were considered significant (*P* < 0.05) between the groups. RT-PCR analysis revealed significant changes between groups in several genes implicated in a variety of disorders ranging from PTSD, anxiety, mood disorders, and substance dependence.

## 1. Background

Posttraumatic stress disorder (PTSD) is characterized by the occurrence of a traumatic event that is beyond the normal range of human experience, for example, rape, torture, genocide, and war zone stress [1]. The range of symptoms of PTSD necessitates many different therapeutic approaches to treating the disorder. The most successful treatments are cognitive-behavioral therapy (CBT) and medications [1]. The selective serotonin reuptake inhibitors (SSRIs), sertraline (Zoloft) and paroxetine (Paxil), are approved by the FDA for treatment of PTSD [1]. The future of PTSD treatment may include medications specifically targeted for the molecular mechanisms responsible for PTSD symptoms. Zhang and colleagues used microarray technology to compare the gene expression profile of stressed maladapted rats (PTSD) and stressed well adapted rats in the areas of the brain associated with PTSD, the hippocampus, and the amygdala [2]. In the hippocampus they found about 110 genes were upregulated by a two-fold change or greater, and about 240 genes were downregulated by less than a half-fold change [2]. They found that multiple two signaling pathways were upregulated and multiple one signaling pathways were downregulated in the amygdala [2].

In the US, nearly 1 in 5 adults reports taking herbal products to treat a medical illness; in fact, as recently as 1980, the US Pharmacopeia consisted of 59% herbal medicines [3]. Green tea is an herbal product that has been consumed since ancient times to promote relaxation. L-theanine (marketed by the trade name Suntheanine) is the amino acid in green tea primarily responsible for its flavor and pharmacologic effect [4]. It was discovered in 1949 and is found almost solely in green tea leaves [4]. In a small-scale human study by Junega and colleagues, Suntheanine was found to promote the generation of alpha brain waves, indicative of a relaxed state. L-theanine is a derivative of L-glutamic acid, which is one of the major excitatory neurotransmitters in the brain [5].

Although little is known about the pharmacokinetics of L-theanine in humans, animal studies show that it is absorbed in the small intestine via sodium-coupled active transport, crosses the blood brain barrier via a leucine-preferring transport system, and is metabolized by the kidneys [5]. In rats, an intraperitoneal injection of L-theanine is taken up by the brain within thirty minutes without metabolic changes [4].

In addition to the study by Junega and colleagues, other researchers have investigated the ability of L-theanine to attenuate anxiety. Lu and colleagues compared the acute effects of L-theanine, alprazolam (a prototype anxiolytic), and a placebo on the behavioral measures of anxiety in healthy human subjects using an anticipatory anxiety model. Lu and colleagues found that L-theanine may have a relaxing effect under resting conditions, but neither L-theanine nor alprazolam had anxiolytic effects in the anticipatory anxiety model [6]. However, in a rat study by Heese et al., L-theanine concomitantly administered with midazolam was shown to have an interactive anxiolytic effect, and a decrease in fine and basic motor movements in a rodent model [7].

No studies have evaluated the potential therapeutic properties of herbal medications in PTSD or changes in gene expression. Because PTSD is categorized as an anxiety or nervous disorder, it is important to study the potential benefits of L-theanine in the treatment of PTSD and the molecular pathways responsible. The purpose of this study was to investigate if L-theanine modulates gene expression in the hippocampus and amygdala, regions of the brain that have been identified as critically important areas involved in the pathogenesis of PTSD [8–10]. This study was part of a larger study that also investigated neurobehavioral changes in this rodent model. This current investigation provides a beginning framework to pursue targeted treatments of PTSD by identifying genes involved in the neurobiology of this devastating condition.

## 2. Methods

Eighty male Sprague-Dawley rats (Harlan Sprague-Dawley Laboratories) weighing 300–350 grams were used. They were housed in groups of three in polycarbonate “shoebox-sized” cages lined with bedding. The animals went through a 7-day adaptation period in a temperature-controlled environment (22 ± 1°C, 60% humidity) with a reverse light-dark cycle where they received 12 hours of light (6:00 PM to 06:00 AM) and 12 hours of darkness (6:00 AM to 06:00 PM). They were allowed free access to food and water. The animals were only handled for weighing, drug administration, and cleaning of cages and were naïve to all instruments. After acclimation, the investigators measured baseline body weight. Statistical analysis demonstrated equivalency between the control and PTSD-stressed groups. The use of laboratory rats in this protocol was in accordance with the NIH Guide for the Care and Use of Laboratory Animals and received approval from the Institutional Animal Care and Use Committee (IACUC) at the US Army Institute of Surgical Research, San Antonio, Texas.

The animals were randomly assigned to one of eight groups by use of a computer number generator. Rats were assigned to the nonstressed groups or the 3-day restraint shock PTSD rodent model groups. There were four groups in the nonstressed rats: control vehicle (C-V), L-theanine (C-L), midazolam (C-M), and L-theanine + midazolam (C-M + L). There were also four groups in the PTSD-induced rats: control vehicle (P-V), L-theanine (P-L), midazolam (P-M), and L-theanine + midazolam (P-M + L). Each animal received a subcutaneous injection of the following: (1) control (saline); (2) L-theanine 10 mg/kg [11]; (3) midazolam (Versed) 1.5 mg/kg; or (4) L-theanine 10 mg/kg + midazolam (Versed) 1.5 mg/kg (to evaluate interaction effects). The vehicle (saline) group served as a control. All animals received equivalent volumes of subcutaneous injection to total 2 mL/kg body weight. Additionally, all experimentation occurred on a timed schedule between 8 AM and 4 PM over 4 consecutive days to control for the circadian rhythm of the animals.

### 2.1. PTSD Stress Model

Stress exposure consisted of 2-hour sessions per day of immobilization and tail-shocks for 3 consecutive days. The animals were stressed in the morning (between 08:00 AM and 12:00 PM). They were restrained in a plexiglass tube, and 40 electric shocks (2 mA, 3-second duration) were applied at varying intervals (140–180 seconds). Commercial hardware and software controlled the timing and amplitude of the shock (Precision Animal Shocker, Coulbourn Instruments, Columbus, Ohio, USA). Rats were stressed for 3 consecutive days. The rationale for the approach was because it had been previously demonstrated that repeated stress sessions for 3 days were more effective than a single stress session in producing sustained physiological and behavioral abnormalities, such as exaggerated acoustic startle response and reduced body weight [12].

Several laboratory studies have established and tested the inescapable tail-shock model of stress in rats. These tests have verified the neurobiological and behavioral alterations induced in the rat model and are similar to those found in human PTSD patients [12, 13]. Rats were stressed for three consecutive days based on previous literature which indicated repeated stress sessions were more effective than a single stress session in producing physiological and behavioral abnormalities, including exaggerated ASR and reduced body weight (see Table 1) [12].

### 2.2. Gene Analysis

**Figure 1:**
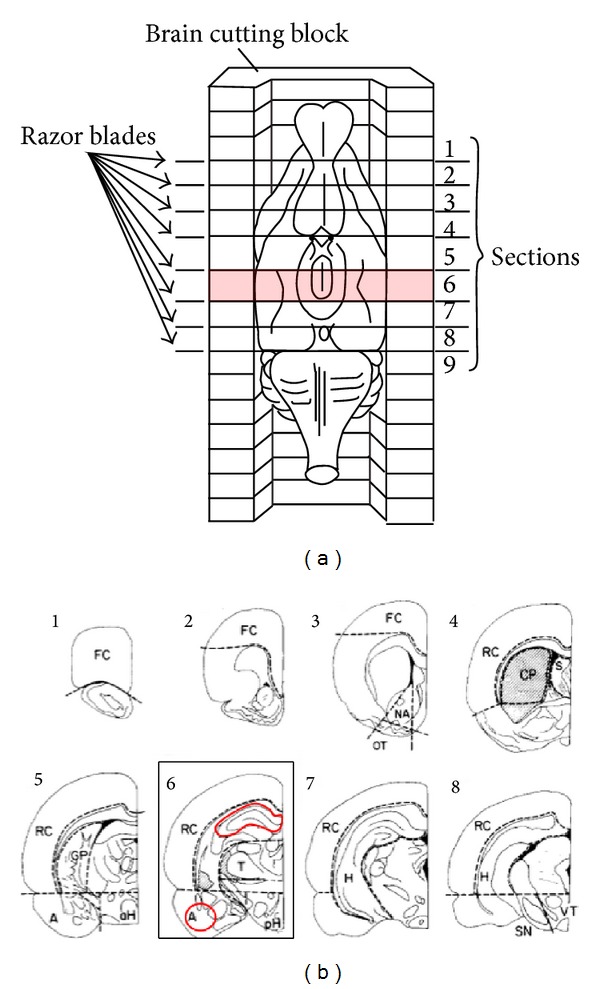
(a) Orientation of brain within brain cutting block; (b) coronal brain sections from dissected regions. (a) Illustration of rat brain in cutting block depicting specific areas where coronal slices were performed; (b) numbers correspond to dissected sections from (b). FC, frontal cortex; NA, nucleus accumbens; OT, olfactory tubercle; S, septum; CP, caudate putamen; RC, remaining cortex; GP, globus pallidus; aH, anterior hypothalamus; pH, posterior hypothalamus; A, amygdala; T, thalamus; SN, substantia nigra; VT, ventral tegmentum; H, hippocampus [14].

After the completion of neurobehavioral tests, the investigators anesthetized the male Sprague-Dawley rats using isoflurane in a bell jar, and the calvarium was removed exposing the whole brain. The whole brain was removed intactly and placed immediately on a brain block on ice [14]. Coronal slices (200 *μ*m thickness) in the vicinity of interaural and bregma coordinates 6.96 mm and −2.04, respectively, were dissected using a brain block (see red shaded region in Figure 1(a)). Circular punches (80–100 *μ*m diameter) were retrieved from the left and right amygdala (see lower left red circle in area 6 of Figure 1(b)). Following the dissection of the amygdala, the investigator grossly dissected the bilateral hippocampi (see upper medial red outlined area in area 6 of Figure 1(b)). Tissue was snap-frozen in liquid nitrogen, placed in prelabeled eppendorf tubes, and packed in dry ice for shipping to the QIAGEN Service Core for Genomics and Gene Expression in Frederick, MD.

RNA was isolated using the QIAGEN RNEasy Mini Kit (Cat. no. 74104) following the manufacturer's protocol for tissue. RNA quality was determined using the Agilent Bioanalyzer (Agilent) with RNA 6000 Nano Kits (Agilent, Cat. no. 5067-1511). Total RNA yield, 260/280, and 260/230 ratios were measured using a NanoDrop spectrophotometer (Thermo). A reverse transcription reaction using 500 ng of total RNA was completed using the QIAGEN RT^2^ First Strand Kit (QIAGEN, Cat. no. 330401). cDNA samples were assayed using a modified QIAGEN RT^2^ PCR array (Cat. no. PARN-60) according to manufacturer's instructions [15, 16]. This array, containing 84 assays related to rat neurotransmitter receptors and regulators, was modified to include four additional assays of genes from QIAGEN. These genes are related to neurotransmission pathways that have been identified in previous PTSD studies: p11 (S100a10) (Cat. no. PPR06766) [17]; 5HT_2A_ receptor (Htr2a) (Cat. no. PPR06850) [18]; alpha_1_ adrenergic receptor (Adra1a) (Cat. no. PPR43329) [19–21]; and Egr1 (Zif/268) (Cat. no. PPR44272) an effector immediate early gene [15, 16].

### 2.3. Rat Neurotransmitter Receptors and Regulators PCR Array

The rat neurotransmitter receptors and regulators RT^2^ profiler PCR array profiles the expression of 84 genes involved in modulating the biological processes of neurotransmitter biosynthesis, uptake, transport, and signaling through neurotransmitter receptors. This array contains receptors for specific neurotransmitters, such as acetylcholine, benzodiazepine, dopamine, gamma-aminobutyric acid (GABA), glutamate, serotonin, somatostatin, and neuropeptides. Genes involved in the regulation of neurotransmitter levels were included as well. Analysis of the expression of a focused panel of genes related to neuronal systems with this array can be performed using real-time PCR.

### 2.4. Statistical Analyses

For this cross-sectional, randomized, prospective study, a one-way ANOVA was performed to compare the 8 groups, of which each unit was randomly allocated. All assumptions were examined (e.g., homogeneity of variance, univariate normality, etc.) and in the event that the assumptions are not tenable remedial (e.g., transformation) or alternative (e.g., nonparametric tests, such as Kruskal-Wallis) strategies were considered. Multiple comparison procedures (MCP) were performed in the event that significance (*α* = .05) was obtained, with the Tukey post hoc test. A variance explained statistic eta-squared (*η* ^2^) was used as the reported effect size, and though ascertaining what constitutes a small/medium/large effect is primarily a function of the context/discipline, for this report .01/.059/.138 was small/medium/large [22]. All data were statistically analyzed based on gene cycle thresholds (CTs) normalized to five housekeeping genes per QIAGEN procedures [15, 16].

## 3. Results

**Figure 2:**
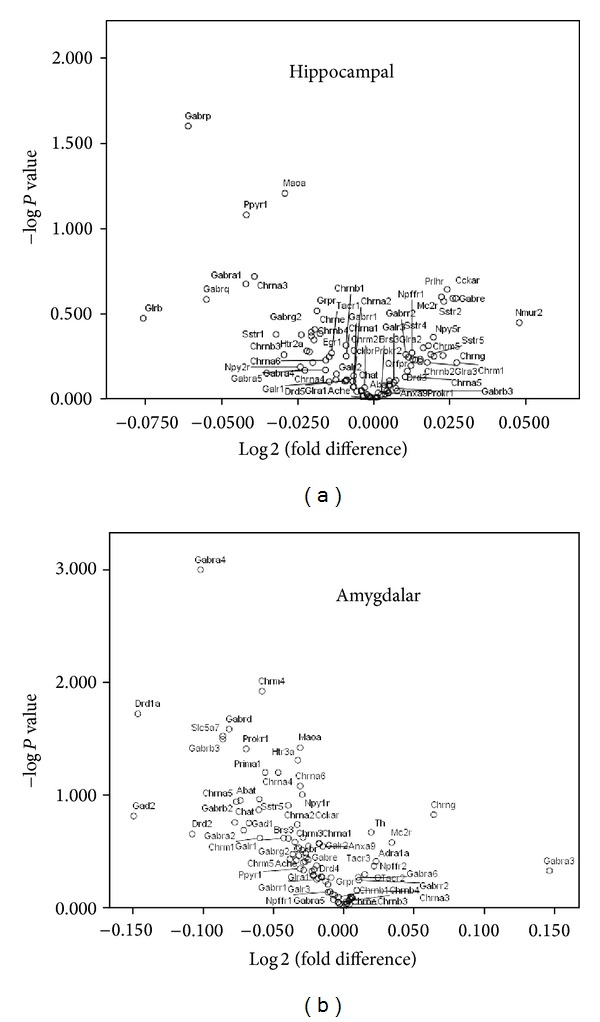
Differential expression of (a) hippocampal and (b) amygdalar mRNA between groups. Volcano plot between Log 2 fold change on the *x*-axis (for neurotransmitter receptors and regulators in L-theanine/midazolam treated groups 40 PTSD-stressed versus 40 nonstressed (control) groups) and −log of *P* value on the *y*-axis. Eighty male Sprague-Dawley rats were injected subcutaneously 30 minutes prior to evaluation of their performance on neurobehavioral tests. The rats were then euthanized and cDNA prepared from the hippocampus (a) and amygdala (b) and were subjected to RT^2^ profiler PCR array for rat neurotransmitter receptor and regulator analysis, as described in Materials and Methods. PCR array profiles were performed for the expression of 88 genes potentially involved in PTSD and/or rat neurotransmitter receptors and neurotransmitter regulation.

This study was designed to investigate the effects of L-theanine on PTSD-induced changes in rat brain gene expression. Eighty-eight genes were investigated in this study. Data analysis showed a number of significant differences in gene expression in both the hippocampus and amygdala. Volcano plots were developed to compare the gene expression of the 40 nonstressed to the 40 PTSD-stressed rats for both the hippocampus and amygdala as demonstrated in Figure 2. The volcano plots graphically display the relationship of –log 10 *P* value and the Log 2 fold change for the combined treatment and control groups for all of the genes. For the 8-group one-way ANOVA, a large effect size (*η* ^2^ > .138) was chosen to serve as a springboard for discussion and interpretation of relative between-group differences on gene expression.

### 3.1. Hippocampus

**Figure 3:**
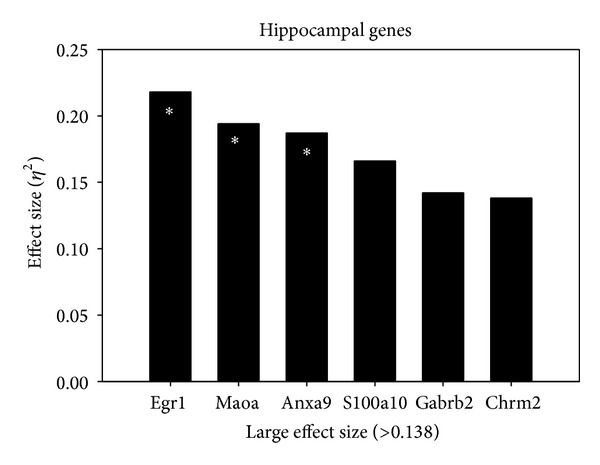
Effect size of gene changes in the hippocampus. Genes demonstrating a large effect size (*η* ^2^ > .138) in the hippocampus as described in the Statistical Analysis.  *Significant *P* value < .05.

In the hippocampus, the genes with an effect size > 0.138 are displayed in Figure 3. In accordance with Figure 3, Table 2 summarizes between-group changes in gene expression in the hippocampus. The Egr1 gene was found to be significant and between-group differences were obtained: *P* = .011, *η* ^2^ = .218. There was one significant post hoc test with the P-M group having a significantly higher mean (*M* = 5.58) than the P-V group (*M* = 4.57). A significant difference was found between groups for the Maoa gene: *P* = .024, *η* ^2^ = .194. The post hoc test found one significant pairwise comparison: (1) the C-M + L group had the highest mean (*M* = 3.32) and the P-V group had the lowest (*M* = 3.12). The Anxa9 gene was found to have significance between groups: *P* = .031, *η* ^2^ = .187. Multiple comparison tests resulted in one significant pairwise result: the P-M group had a significantly higher mean (*M* = 10.73) than the P-V group (*M* = 10.06).

The following genes in the hippocampus did not show significant differences between groups; however, due to the large effect size (variance explained) these genes are presented in Table 2. The S100a10 gene did not have significant between-group differences: *P* = .06, *η* ^2^ = .166. The P-M group had the highest mean (*M* = 4.2) and the P-V group had the lowest (*M* = 3.82). Significant between-group differences were not obtained for the Gabrb2 gene: *P* = .123, *η* ^2^ = .142. The P-M group had the highest mean (*M* = 3.32) and the P-V group had the lowest (*M* = 2.75). Finally, the Chrm2 gene did not show significance between groups. A statistically significant difference was not obtained: *P* = .138, *η* ^2^ = .138. Though not significant, the C-M + L group had the highest mean (*M* = 10.56) and the P-V group had the lowest (*M* = 9.96).

### 3.2. Amygdala

**Figure 4:**
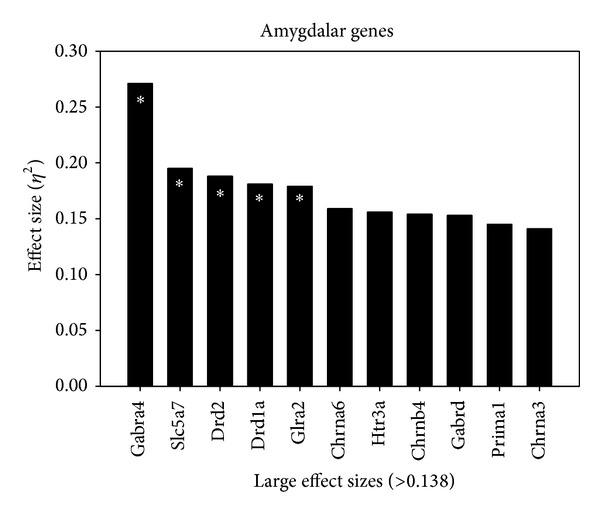
Effect size of gene changes in the amygdala. Genes demonstrating a large effect size (*η* ^2^ > .138) in the amygdala as described in the Statistical Analysis.  *Significant *P* value < .05.

Genes in the amygdala with an effect size greater than 0.138 are displayed in Figure 4. In accordance with Figure 4, Table 3 summarizes between-group differences in gene expression within the amygdala. The Gabra4 gene was *P* = .001, *η* ^2^ = .271. Significant post hoc pairwise comparisons were such that the C-L group (*M* = 3.52) had a higher mean than each of the following: (1) P-L (*M* = 3.04), (2) P-M (*M* = 3.11), and (3) C-M group (*M* = 3.09). A significant difference for the Slc5a7 gene was found: *P* = .024, *η* ^2^ = .195. The C-V group had the highest mean (*M* = 7.75) and the P-L group had the lowest (*M* = 6.67); however as per the post hoc test, this pairwise comparison was not significant. Significant between-group differences were also found for the Drd2 gene: *P* = .02, *η* ^2^ = .189. There was a significant difference between P-V (*M* = 6.76) and the P-M group (*M* = 4.48). The Drd1a gene was also significant: *P* = .038, *η* ^2^ = .181. A significant difference was found between the C-M + L (*M* = 6.41) and the P-M group (*M* = 4.83). Lastly, the Glra2 showed significance: *P* = .04, *η* ^2^ = .179. There was one significant post hoc test between the C-M + L group with the higher mean (*M* = 5.62) and the P-M group with the lower mean (*M* = 4.89).

Again the following genes were not found to be significant; however, based on the large effect size (variability) used in the study these genes are included in Table 3. No difference was found for the Chrna6 gene: *P* = .075, *η* ^2^ = .159. The C-M group had the highest mean (*M* = 16.51) and the C-M + L group had the lowest (*M* = 15.5). The Htr3a gene showed no significant difference between the groups: *P* = .081, *η* ^2^ = .156. The C-L group had the highest mean (*M* = 6.74) and the P-M + L group had the lowest (*M* = 6.35). The Chrnb4 also showed no significant difference: *P* = .087, *η* ^2^ = .154. The C-V group had the highest mean (*M* = 11.59) and the C-M + L group had the lowest (*M* = 10.43). No significant difference was found for the Gabrd gene between the groups: *P* = .089, *η* ^2^ = .153. The C-M + L group had the highest mean (*M* = 5.23) and the P-M group had the lowest (*M* = 4.56). The Prima1 gene did not have a significant difference between the groups: *P* = .111, *η* ^2^ = .145. The P-V group had the highest mean (*M* = 6.94) and the P-M group had the lowest (*M* = 6.25). No difference between the groups was found for the Chrna3 gene: *P* = .127, *η* ^2^ = .141. The P-L group had the highest mean (*M* = 10.56) and the C-M + L group had the lowest (*M* = 9.67).

## 4. Discussion

L-theanine has previously been demonstrated to produce anxiolytic effects alone or in combination with concomitant administered benzodiazepines [7]. No studies have examined the administration of L-theanine's effects on PTSD-induced changes in brain gene expression. We explored the modulation of gene expression in PTSD-induced changes in the hippocampus and amygdala and the effects of L-theanine and midazolam on these changes.

### 4.1. Hippocampus

We found a downregulation of the Egr1 gene in the PTSD-vehicle group compared to the PTSD-midazolam group. Egr1 is an early growth response nuclear transcription factor that plays a role in regulating the expression of genes required for learning and memory [23, 24]. In particular, previous studies have demonstrated a role for Egr1 in hippocampal long-term potentiation, a process critical to learning and memory [25]. Expression of the Maoa gene was decreased in the PTSD-vehicle group compared to the control group that received L-theanine and midazolam.

Decreased Maoa enzymatic activity leads to increased concentrations of its substrate biogenic amines, including norepinephrine and serotonin. As reviewed by Bortolato and colleagues, previous research has demonstrated strong links between decreased Maoa activity and a number of reactive social behaviors, including specific links to violence and aggression [26]. Similarly, decreased expression of Maoa in humans predicted, among other changes, amygdala and hippocampus hyperreactivity during aversive recall [27].

Although no significant difference was found between groups, the PTSD-vehicle group had the lowest mean expression of the S100a10 gene and the PTSD-midazolam group the highest. The S100a10 gene and its associated protein p11 are important in the regulation of serotonin receptors [28], and decreases in S100a10 gene expression have been linked to PTSD, major depressive disorder, and bipolar disorder and proposed as a likely biomarker for suicidality [29, 30].

A downregulation of the Gabrb2 gene in the PTSD-vehicle group compared to the PTSD-midazolam group was similar to Zhao and colleagues findings of a significantly decreased expression of the Gabrb2 gene in the anterior cingulate cortex in subjects suffering from major depressive disorder or bipolar disorder [31].

A downregulation of the Anxa9 gene was found in the PTSD-vehicle group compared to the PTSD-midazolam group. The Anxa9 gene, part of the calcium-dependent phospholipid-binding annexin family, may play a role in suppression of apoptosis and is currently under investigation as a therapeutic target for the treatment of breast cancer [32]. In a study using the Flinders rat model, Chrm2 gene expression levels were found to be significantly lower in the Flinders depression sensitive line [33].

### 4.2. Amygdala

According to Ponomarev and colleagues, downregulation of the Gabra4 gene can be correlated with stress enhanced fear learning (SEFL) in rats. These data suggest a significant role of GABA signaling in SEFL. The method of inducing SEFL was similar to the method of inducing PTSD in our study [34]. Interestingly, our findings suggest an upregulation of this gene in the nonstressed-L-theanine group compared to PTSD-L-theanine, PTSD-midazolam, and nonstressed-midazolam groups.

PCR analysis of tissue samples from the amygdala showed a 2.28-fold increase in Drd2 gene expression in the PTSD-vehicle group compared to the PTSD-midazolam group, which is inconsistent with the findings of a study that investigated the effects of maternal deprivation and chronic mild stress in rats [35]. Zhu and colleagues showed that mRNA levels of the Drd2 gene were significantly decreased in all stressed groups compared to controls, which enabled the investigators to correlate a downregulation of the Drd2 gene with depressive phenotypes [35].

Drd1a genes were significantly downregulated in the PTSD-midazolam group compared to the nonstressed-midazolam + L-theanine group concurrent with the results of another study exploring the effects of stress on the mouse prefrontal cortex transcriptome, demonstrating a deregulation of the Drd1a gene in chronic mild stress exposed subjects [36].

The SLc5a7 gene was significantly downregulated in the PTSD-L-theanine group compared to the nonstressed-vehicle group. In comparison the SLc5a7 gene was found to be upregulated in tasks that required higher levels of attention, and it was found that decreased levels of cholinergic activity are involved in ADHD [37].

Prima1 DNA methylation increased by 10–15% in subjects suffering from major depressive disorder [38] which interestingly shows some correlation with our finding of the highest level of Prima1 gene expression in the PTSD-vehicle group, which is a 0.69-fold increase in expression compared with the PTSD-midazolam group with the lowest level of expression.

Although no studies have investigated gene expression changes of Chrnb4 in PTSD or anxiety, variations in this gene were shown to increase sensitivity to nicotine's effects on attention, mood, and vulnerability to tobacco dependence [39]. The Htr3a gene and serotonin have been linked to anxiety and moods as well as psychoses [40], and interactions with 5HT3 receptors are also involved with aggressive behavior, depression, and the stress response [41]. The Gabrd gene is a potential susceptibility gene for childhood-onset mood disorders [42], and variations in this gene are associated with a range of genetic epilepsy syndromes [43] including febrile seizures [44]. Chrna6 and Chrna3 genes are involved in dopaminergic neurotransmission, specifically noted in mesodiencephalic dopamine releasing neurons [45]. At this time, there is no sufficient research linking the Glra2 gene with PTSD, anxiety, or mood disorders.

## 5. Conclusions

RT-PCR analysis revealed significant changes between groups in several genes implicated in a variety of disorders ranging from PTSD, anxiety, mood disorders, and substance dependence. These data further elucidate the transcriptomic footprint of PTSD in the rodent amygdala and hippocampus as well as transcriptome changes effected by L-theanine and midazolam interventions.

These different neurotransmitter genes are significant biologically and clinically as they are associated with various central nervous system pathologies. The hippocampal genes related to long-term potentiation (Egr1), violence and aggression (Maoa), and major depressive and bipolar disorder (S100a10, serotonin receptors) were shown to be downregulated in PTSD animals and expression levels significantly changed. Other neurotransmitter genes Gabrb2 and Chrm2 were also modulated across treatment groups and warrant further exploration as they are associated with depression related clinical conditions. Amygdalar genes that were changed across treatment groups also were related to psychological conditions such as depression (Drd2), stress enhanced fear learning (Gabra4), chronic mild stress (Drd1a), and anxiety, mood disorders, and psychoses (Htr3a). These findings taken together demonstrate a relationship of possible gene expression changes in important and known neurotransmitter system genes and associated clinical pathologies. This study lays the foundation for future studies to interrogate and investigate the translation of these genes into proteins and the potential for these proteins to act as sites for pharmacologic therapeutic intervention.

This understanding of the effect of PTSD at the level of mRNA transcription contributes to a more complete understanding of the overall metabonomics of PTSD and may allow for more targeted pharmacologic intervention in the future.

The next step to consider in clarifying the molecular mechanism of PTSD may be to characterize PTSD-induced changes to the rat proteome. Changes in mRNA expression are often linked to changes in protein expression, but not in a predictable manner. Small changes in mRNA expression can lead to variable changes in corresponding protein expression. We propose repeating the current experiment using protein assay methods to correlate changes in mRNA expression with changes in protein expression induced by PTSD and the effects of L-theanine and midazolam on these changes. Multiple methods and computational approaches to protein assays are available [46].

We anticipate further investigation into the network of gene relationships between control and PTSD-induced rats using bioinformatic methods such as gene enrichment analysis and network enrichment analysis. Characterization of a comprehensive proteomic network in both control and PTSD-induced rats will allow for comparative proteomic analysis that may lead to the identification of biomarkers for PTSD susceptibility as well as potential targets for pharmacologic interventions [47–49]. Additionally, we anticipate investigation into the chemical components of other herbals or pharmaceuticals that may have similar effects on gene expression at the level of mRNA transcription, with a particular emphasis on those with demonstrated efficacy in treating PTSD.

## Figures and Tables

**Table 1:** Comparison of symptoms of PTSD in humans to dysfunction related to stress in rats (restraint/shock) [12, 13].

PTSD in humans	Rat inescapable tail-shock model of stress (3 days)
Weight loss	Suppressed feeding and body weight loss
Difficulty falling or staying asleep, nightmares	Altered sleep patterns
Psychomotor numbness	Persistent behavioral abnormalities, that is, suppressed open-field activity, longer hanging wire latencies
Poor concentration; memory deficits	Deficits in escape/avoidance learning and learning of an appetitive task
Hyperarousal/startle response	Exaggerated startle response

**Table 2:** Between-group changes in gene expression within the hippocampus.

Gene	^ #^Group comparison	Fold change	Effect size	*P* value	Description of gene
Egr1	P-M versus P-V	1.01	0.218	∗.011	Long-term memory formation [50]

Maoa	C-M + L versus P-V	0.2	0.194	∗.024	Degrades biogenic amines, including catecholamines and serotonin [51]

Anxa9	P-M versus P-V	0.67	0.187	∗.031	An atypical member of the annexin family of Ca^2+^ and phospholipid-binding proteins, responsible for Ca^2+^ coordination [52]

S100a10	P-M versus P-V	0.38	0.166	.06	Intracellular p11 (S100a10) regulates serotonin receptor activity in psychiatric mood disorders [53]. Potential biomarker for PTSD [30] modulated by glucocorticoids [17]

Gabrb2	P-M versus P-V	0.57	0.142	.123	A subunit of GABA type-A receptors, essential for fast inhibitory neurotransmission, critical in brain function [54]

Chrm2	C-M + L versus P-V	0.6	0.138	.138	A gene implicated in self-regulatory processes across a range of externalizing behaviors [55]

^#^Group comparison column presents the highest and lowest mean; ∗significant *P* value < .05. PTSD-vehicle (P-V); PTSD-midazolam (P-M); PTSD-L-theanine (P-L); PTSD-midazolam + L-theanine (P-M + L); control vehicle (C-V); control midazolam (C-M); control L-theanine (C-L); control midazolam + L-theanine (C-M + L); large effect size > 0.138; significant *P* value < .05. All genes were confirmed via the National Center for Biotechnology Information (NCBI) database [56].

**Table 3:** Between-group changes in gene expression within the amygdala.

Gene	^ #^Group comparison	Fold change	Effect size	*P* value	Description of gene
Gabra4	C-L versus P-L	0.48	0.271	∗.001	A subunit of GABA type-A receptors responsible for encoding GABA [57]
	C-L versus P-M	0.41			
	C-L versus C-M	0.43			

SLc5a7	C-V versus P-L	1.08	0.195	∗.024	A Na^+^ and Cl^−^ transporter that mediates the uptake of choline for ACh synthesis in cholinergic neurons [58]

Drd2	P-V versus P-M	2.28	0.188	∗.03	Effect of mRNA stability and synthesis of the type 2 dopamine receptor [59]

Drd1a	C-M + L versus P-M	1.58	0.181	∗.038	Plays a fundamental role in spatial working memory and brain-derived neurotrophic factor (BDNF) expression in prefrontal cortex [60]

Glra2	C-M versus C-M + L	0.73	0.179	∗.04	Provides instructions for making the alpha-1 subunit of the glycine receptor protein [61]

Chrna6	C-M versus C-M + L	1.01	0.159	.075	Codes for the alpha-6 subunit found in certain types of nicotinic acetylcholine receptors [62]

Htr3a	C-L versus P-M + L	0.39	0.156	.081	Gene encodes subunit A of the type 3 receptor for 5-HT (serotonin) [63]

Chrnb4	C-V versus C-M + L	1.16	0.154	.087	A nicotinic ACh receptor gene subtype. It is partially responsible for ETOH and tobacco addiction [64]

Gabrd	C-M + L versus P-M	0.67	0.153	.089	This gene encodes the delta subunit of GABA [65]

Prima1	P-V versus P-M	0.69	0.145	.111	Functions to organize acetylcholinesterase (AChE) into tetramers and to anchor AChE at neural cell membranes [66]

Chrna3	P-L versus C-M + L	0.89	0.141	.127	Encodes an alpha-type subunit, in the nicotinic acetylcholine receptor [67]

^#^Group comparison column presents the highest and lowest mean; ∗significant *P* value < .05. PTSD-vehicle (P-V); PTSD midazolam (P-M); PTSD-L-theanine (P-L); PTSD midazolam + L-theanine (P-M + L); control vehicle (C-V); control midazolam (C-M); control L-theanine (C-L); control midazolam + L-theanine (C-M + L); large effect size > 0.138; significant *P* value < .05. All genes were confirmed via the National Center for Biotechnology Information (NCBI) database [56].

## References

[1] Friedman M (2007). *PTSD History and Overview*.

[2] Zhang L, Zhou R, Xing G, Hough CJ, Li X, Li H (2006). Identification of gene markers based on well validated and subcategorized stressed animals for potential clinical applications in PTSD. *Medical Hypotheses*.

[3] Bent S (2008). Herbal medicine in the United States: review of efficacy, safety, and regulation: grand Rounds at University of California, San Francisco Medical Center. *Journal of General Internal Medicine*.

[4] Chu D, Okubo T, Nagato Y, Yokogoshi H (1999). L-theanine—a unique amino acid of green tea and its relaxation effect in humans. *Trends in Food Science and Technology*.

[5] Eschenauer G, Sweet BV (2006). Pharmacology and therapeutic uses of theanine. *The American Journal of Health-System Pharmacy*.

[6] Lu K, Gray MA, Oliver C (2004). The acute effects of L-theanine in comparison with alprazolam on anticipatory anxiety in humans. *Human Psychopharmacology*.

[7] Heese T, Jenkinson J, Love C (2009). Anxiolytic effects of L-theanine—a component of green tea-when combined with midazolam, in the male Sprague-Dawley rat. *AANA Journal*.

[8] Liberzon I, Martis B (2006). Neuroimaging studies of emotional responses in PTSD. *Annals of the New York Academy of Sciences*.

[9] Pitman RK, Shin LM, Rauch SL (2001). Investigating the pathogenesis of posttraumatic stress disorder with neuroimaging. *Journal of Clinical Psychiatry*.

[10] Ursano RJ, Zhang L, Li H (2009). PTSD and traumatic stress: from gene to community and bench to bedside. *Brain Research*.

[11] Sugiyama T, Sadzuka Y (1999). Combination of theanine with doxorubicin inhibits hepatic metastasis of M5076 ovarian sarcoma. *Clinical Cancer Research*.

[12] Servatius RJ, Ottenweller JE, Natelson BH (1995). Delayed startle sensitization distinguishes rats exposed to one or three stress sessions: further evidence toward an animal model of PTSD. *Biological Psychiatry*.

[13] Garrick T, Morrow N, Shalev AY, Eth S (2001). Stress-induced enhancement of auditory startle: an animal model of posttraumatic stress disorder. *Psychiatry*.

[14] Heffner TG, Hartman JA, Seiden LS (1980). A rapid method for the regional dissection of the rat brain. *Pharmacology Biochemistry and Behavior*.

[15] Kozlovsky N, Matar MA, Kaplan Z, Zohar J, Cohen H (2009). A distinct pattern of intracellular glucocorticoid-related responses is associated with extreme behavioral response to stress in an animal model of post-traumatic stress disorder. *European Neuropsychopharmacology*.

[16] *Rat Neurotransmitter Receptors and Regulators PCR Array*.

[17] Zhang L, Li H, Su TP (2008). p11 is up-regulated in the forebrain of stressed rats by glucocorticoid acting via two specific glucocorticoid response elements in the p11 promoter. *Neuroscience*.

[18] Jiang X, Xing G, Yang C, Verma A, Zhang L, Li H (2009). Stress impairs 5-HT_2A_ receptor-mediated serotonergic facilitation of GABA release in juvenile rat basolateral amygdala. *Neuropsychopharmacology*.

[19] Manion ST, Gamble EH, Li H (2007). Prazosin administered prior to inescapable stressor blocks subsequent exaggeration of acoustic startle response in rats. *Pharmacology Biochemistry and Behavior*.

[20] Miller LJ (2008). Prazosin for the treatment of posttraumatic stress disorder sleep disturbances. *Pharmacotherapy*.

[21] Raskind MA, Peskind ER, Hoff DJ (2007). A parallel group placebo controlled study of prazosin for trauma nightmares and sleep disturbance in combat veterans with post-traumatic stress disorder. *Biological Psychiatry*.

[22] Cohen J (1988). *Statistical Power Analysis for the Behavioral Sciences*.

[23] Davis S, Bozon B, Laroche S (2003). How necessary is the activation of the immediate early gene *zif268* in synaptic plasticity and learning?. *Behavioural Brain Research*.

[24] Knapska E, Kaczmarek L (2004). A gene for neuronal plasticity in the mammalian brain: Zif268/Egr-1/NGFI-A/ Krox-24/TIS8/ZENK?. *Progress in Neurobiology*.

[25] Penke Z, Chagneau C, Laroche S (2011). Contribution of Egr1/Zif268 to activity-dependent Arc/Arg3.1 transcription in the dentate gyrus and area CA1 of the hippocampus. *Frontiers in Behavioral Neuroscience*.

[26] Bortolato M, Chen K, Godar SC (2011). Social deficits and perseverative behaviors, but not overt aggression, in MAO-A hypomorphic mice. *Neuropsychopharmacology*.

[27] Meyer-Lindenberg A, Buckholtz JW, Kolachana B (2006). Neural mechanisms of genetic risk for impulsivity and violence in humans. *Proceedings of the National Academy of Sciences of the United States of America*.

[28] Svenningsson P, Chergui K, Rachleff I (2006). Alterations in 5-HT1B receptor function by p11 in depression-like states. *Science*.

[29] Su T-P, Zhang L, Chung M-Y (2009). Levels of the potential biomarker p11 in peripheral blood cells distinguish patients with PTSD from those with other major psychiatric disorders. *Journal of Psychiatric Research*.

[30] Zhang L, Su T, Choi K (2011). P11 (S100A10) as a potential biomarker of psychiatric patients at risk of suicide. *Journal of Psychiatric Research*.

[31] Zhao J, Bao A-M, Qi X-R (2012). Gene expression of GABA and glutamate pathway markers in the prefrontal cortex of non-suicidal elderly depressed patients. *Journal of Affective Disorders*.

[32] Hu Z, Neve R, Guan Y, Gray J Identification of new therapeutic targets of breast cancer using siRNA.

[33] Blaveri E, Kelly F, Mallei A (2010). Expression profiling of a genetic animal model of depression reveals novel molecular pathways underlying depressive-like behaviours. *PLoS ONE*.

[34] Ponomarev I, Rau V, Eger EI, Harris RA, Fanselow MS (2010). Amygdala transcriptome and cellular mechanisms underlying stress-enhanced fear learning in a rat model of posttraumatic stress disorder. *Neuropsychopharmacology*.

[35] Zhu X, Peng S, Zhang S, Zhang X (2011). Stress-induced depressive behaviors are correlated with Par-4 and DRD2 expression in rat striatum. *Behavioural Brain Research*.

[36] Lisowski P, Wieczorek M, Goscik J (2013). Effects of chronic stress on prefrontal cortex transcriptome in mice displaying different genetic backgrounds. *Journal of Molecular Neuroscience*.

[37] English BA, Hahn MK, Gizer IR (2009). Choline transporter gene variation is associated with attention-deficit hyperactivity disorder. *Journal of Neurodevelopmental Disorders*.

[38] Sabunciyan S, Aryee MJ, Irizarry RA (2012). Genome-wide DNA methylation scan in major depressive disorder. *PloS ONE*.

[39] Picciotto MR, Kenny PJ (2013). Molecular mechanisms underlying behaviors related to nicotine addiction.. *Cold Spring Harbor Perspectives in Medicine*.

[40] Iidaka T, Ozaki N, Matsumoto A (2005). A variant C178T in the regulatory region of the serotonin receptor gene HTR3A modulates neural activation in the human amygdala. *Journal of Neuroscience*.

[41] Popova NK, Naumenko VS (2013). 5-HT1A receptor as a key player in the brain 5-HT system. *Reviews in the Neurosciences*.

[42] Feng Y, Kapornai K, Kiss E (2010). Association of the GABRD gene and childhood-onset mood disorders. *Genes, Brain and Behavior*.

[43] Macdonald RL, Kang J-Q, Gallagher MJ (2010). Mutations in GABA_A_ receptor subunits associated with genetic epilepsies. *The Journal of Physiology*.

[44] Nakayama J (2009). Progress in searching for the febrile seizure susceptibility genes. *Brain and Development*.

[45] Chakrabarty K, von Oerthel L, Hellemons A (2012). Genome wide expression profiling of the mesodiencephalic region identifies novel factors involved in early and late dopaminergic development. *Biology Open*.

[46] Vaudel M, Sickmann A, Martens L (2012). Current methods for global proteome identification. *Expert Review of Proteomics*.

[47] Abatangelo L, Maglietta R, Distaso A (2009). Comparative study of gene set enrichment methods. *BMC Bioinformatics*.

[48] Shojaie A, Michailidis G (2010). Network enrichment analysis in complex experiments. *Statistical Applications in Genetics and Molecular Biology*.

[49] Alexeyenko A, Lee W, Pernemalm M (2012). Network enrichment analysis: extension of gene-set enrichment analysis to gene networks. *BMC Bioinformatics*.

[50] Katche C, Goldin A, Gonzalez C, Bekinschtein P, Medina JH (2012). Maintenance of long-term memory storage is dependent on late posttraining Egr-1 expression. *Neurobiology of Learning and Memory*.

[51] Chen Z-Y, Hotamisligil GS, Huang J-K (1991). Structure of the human gene for monoamine oxidase type A. *Nucleic Acids Research*.

[52] Chlystun M, Markoff A, Gerke V (2004). Structural and functional characterisation of the mouse annexin A9 promoter. *Biochimica et Biophysica Acta—Molecular Cell Research*.

[53] Hedhli N, Falcone DJ, Huang B (2012). The annexin A2/S100A10 system in health and disease: emerging paradigms. *Journal of Biomedicine and Biotechnology*.

[54] Mulligan MK, Wang X, Adler AL, Mozhui K, Lu L, Williams RW (2012). Complex control of GABA(a) receptor subunit mRNA expression: variation, covariation, and genetic regulation. *PLoS ONE*.

[55] Latendresse SJ, Bates JE, Goodnight JA (2011). Differential susceptibility to adolescent externalizing trajectories: examining the interplay between chrm2 and peer group antisocial behavior. *Child Development*.

[56] Gene Database. http://www.ncbi.nlm.nih.gov/gene/.

[57] Mclean PJ, Farb DH, Russek SJ (1995). Mapping of the *α*
_4_ subunit gene (GABRA4) to human chromosome 4 defines an *α*
_2_-*α*
_4_-*β*
_1_-*γ*
_1_ gene cluster: further evidence that modern GABA_A_ receptor gene clusters are derived from an ancestral cluster. *Genomics*.

[58] Apparsundaram S, Ferguson SM, George AL, Blakely RD (2000). Molecular cloning of a human, hemicholinium-3-sensitive choline transporter. *Biochemical and Biophysical Research Communications*.

[59] Duan J, Wainwright MS, Comeron JM (2003). Synonymous mutations in the human dopamine receptor D2 (DRD2) affect mRNA stability and synthesis of the receptor. *Human Molecular Genetics*.

[60] Xing B, Guo J, Meng X, Wei S, Li S (2012). The dopamine D1 but not D3 receptor plays a fundamental role in spatial working memory and BDNF expression in prefrontal cortex of mice. *Behavioural Brain Research*.

[61] Feng J, Craddock N, Jones IR (2001). Systematic screening for mutations in the glycine receptor *α*2 subunit gene (GLRA2)in patients with schizophrenia and other psychiatric diseases. *Psychiatric Genetics*.

[62] Meyer EL, Yoshikami D, McIntosh JM (2008). The neuronal nicotinic acetylcholine receptors *α*4∗ and *α*6∗ differentially modulate dopamine release in mouse striatal slices. *Journal of Neurochemistry*.

[63] Weiss B, Mertz A, Schrok E, Koenen M, Rappold G (1995). Assignment of a human homolog of the mouse Htr3 receptor gene to chromosome 11q23.1-q23.2. *Genomics*.

[64] Gallego X, Cox RJ, Laughlin JR, Stitzel JA, Ehringer MA (2013). Alternative CHRNB4 3′-UTRs mediate the allelic effects of SNP rs1948 on gene expression. *PLoS ONE*.

[65] Sommer B, Poustka A, Spurr NK, Seeburg PH (1990). The murine GABA_A_ receptor *δ*-subunit gene: structure and assignment to human chromosome 1. *DNA and Cell Biology*.

[66] Perrier AL, Massoulié J, Krejci E (2002). PRiMA: the membrane anchor of acetylcholinesterase in the brain. *Neuron*.

[67] Eng CM, Kozak CA, Beaudet AL, Zoghbi HY (1991). Mapping of multiple subunits of the neuronal nicotinic acetylcholine receptor to chromosome 15 in man and chromosome 9 in mouse. *Genomics*.

